# Undeclared pre-exposure or post-exposure prophylaxis (PrEP/PEP) use among syphilis-positive blood donors, England, 2020 to 2021

**DOI:** 10.2807/1560-7917.ES.2023.28.11.2300135

**Published:** 2023-03-16

**Authors:** Victoria Maddox, Claire Reynolds, Alieu Amara, Laura Else, Susan R Brailsford, Saye Khoo, Heli Harvala

**Affiliations:** 1 Microbiology Services, NHS Blood and Transplant, London, United Kingdom; 2 NHS Blood and Transplant/UK Health Security Agency Epidemiology Unit, NHS Blood and Transplant, London, United Kingdom; 3 Department of Pharmacology, University of Liverpool, Liverpool, United Kingdom; 4 National Infection Service, United Kingdom Health Security Agency, London, United Kingdom; 5 Infection and Immunity, University College London, London, United Kingdom

**Keywords:** PrEP, HIV, syphilis, blood donor, blood safety

## Abstract

An individualised blood donor selection policy was implemented in the United Kingdom from summer 2021. We have investigated the impact of this policy by comparing the extent of undeclared use of HIV pre-exposure or post-exposure prophylaxis (PrEP/PEP) before and after this change. The rate of PrEP usage in syphilis-positive male blood donors has not changed since individualised donor assessment was implemented but provides continuing evidence of undisclosed PrEP use which may be associated with current or past higher-risk sexual behaviours.

A more individualised blood donor selection policy was implemented in the United Kingdom (UK) from summer 2021 [1]. It replaced the previous 3-month deferral of men who have sex with men (MSM), and for the first time in the UK, allowed MSM to donate if in a long-term relationship. Pre-exposure prophylaxis (PrEP) is a very effective means to prevent sexual transmission of HIV [2]. It is also considered as a marker of recent likely high-risk sexual activity and can potentially hinder the detection of early HIV infection if not taken as prescribed [3]. Although individuals taking PrEP are deferred from blood donations for 3 months after completion of the course, we have previously found evidence of PrEP use in male blood donors with syphilis infection [4]. To evaluate the impact of this new donor selection policy on blood donation safety, we assessed the extent of PrEP use among syphilis-infected male donors before and after the change.

## Residual plasma samples

This study included 177 anonymised residual plasma samples collected from syphilis-infected male blood donors in England, 2020 to 2021. Routine syphilis testing of all blood donations was performed as described [5]. Anonymised data included donor age, self-reported ethnicity/country of birth, testing results and likely risk factors for infection, the latter obtained from the post-test discussion. A routine post-test discussion aims to inform donors about their syphilis results and the follow-up required. To determine whether they were compliant with donation guidelines, we also asked if they had previously been diagnosed or treated for syphilis, could identify a potential source of infection or had ever taken PrEP.

## Plasma testing for tenofovir and emtricitabine residuals

Plasma samples were tested for tenofovir (TFV) and emtricitabine (FTC) concentration using high-performance liquid chromatography coupled with mass spectrometry [6,7]. The lower limit of quantification was 1 ng/mL for TFV and 5 ng/mL for FTC, and lack of detection of either analyte indicated that no PrEP was taken in the 5 days preceding donation [6]. Plasma TFV concentrations greater than 10 ng/mL would be consistent with PrEP administration within the previous 48–72 h; plasma TFV concentrations of 100 ng/mL have been detected in PrEP users 16 h post dose [6]. As TFV/FTC is also found in post-exposure prophylaxis (PEP) or HIV treatment preparations, it should be noted that without further information from the donors we would be unable to differentiate between them. Donors are asked not to donate if they have HIV or think they need a test for HIV.

## Characteristics of syphilis-infected male donors with PrEP usage in England

Ten donation samples obtained from syphilis-infected male donors (nine with past and one with recent syphilis) were positive for either TFV, FTC or both (Table 1). One of them was estimated to have taken their last TFV/FTC dose within a day of donating, five within 3 days and four within 5 days. At the post-test discussion, one declared a history of PrEP use, and four a previous diagnosis of syphilis. Six donors had previous or current male sexual partners and two had only had female partners, whereas the two remaining donors did not engage in a post-test discussion. The donors were geographically distributed across the UK with four cases in the London area, three cases in or near Manchester and three individual cases elsewhere in England. Most were UK-born (n = 7), followed by two from South America and one from Eastern Europe. The median age of the donors was 41 years (range: 21–53 years; Figure). The finding demonstrates non-compliance with the pre-donation questionnaire, which specifically asks about and excludes donors with a history PrEP use within 3 months [3].

**Table 1:** Characteristics of male blood donors with evidence of recent PrEP use identified in England, January 2020–November 2021 (n = 10)

Donation year	Donation type	Age group	Country/region of birth	Post-test discussion	Male sexual partners	Syphilis status	TFV (ng/mL)	FTC (ng/mL)	Timing of PrEP
2020	First	31–40	Eastern Europe	No further information	Yes	Past	< 1.0	10.33	< 5 days
2020	First	≤ 20	UK	Male and female partner	Yes	Past	< 1.0	16.44	< 5 days
2020	Repeat	41–50	UK	Known past syphilis	No	Past	37.76	74.83	2–3 days
2021	First	31–40	South America	No further information	Yes	Past	95.1	442.29	ca 1 day
2021	First	51–60	UK	Known past syphilis and PrEP use	Yes	Past	43.61	532.77	2–3 days
2021	First	31–40	South America	No response	Not known	Past	59.7	686.14	2–3 days
2021	Repeat	41–50	UK	One-off male partner	Yes	Past	73.97	136.59	2–3 days
2021	First	51–60	UK	Known past syphilis	Yes	Past	86.6	432.31	2–3 days
2021	Repeat	21–30	UK	No response	Not known	Recent^a^	7.3	33.36	< 5 days
2021	First	41–50	UK	Known past syphilis	No	Past	8.82	18.66	< 5 days

TFV: tenofovir; FTC: emtricitabine; PrEP: pre-exposure prophylaxis; UK: United Kingdom.

^a^ Syphilis was defined as recent if it was considered that the donor acquired the infection within 12 months before their last donation based on IgM-positive result or recent symptoms/clinical history indicative of infectious syphilis, or if they were negative on their most recent previous donation within 12 months.

**Figure:**
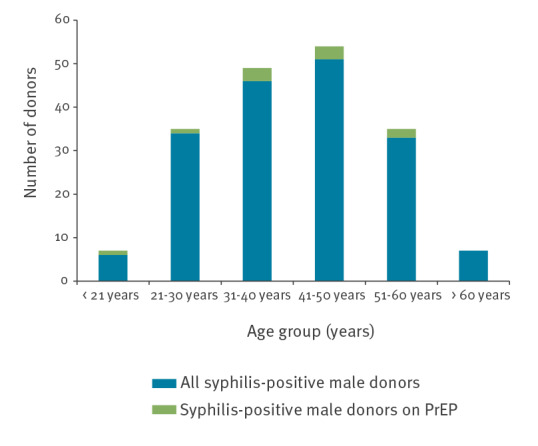
Number of syphilis-positive male blood donors with or without evidence of PrEP, England, January 2020–November 2021 (n = 177)

PrEP: pre-exposure prophylaxis.

## Extent of PrEP use among syphilis-infected male donors between 2020 and 2021

Most syphilis-infected male donors were between 21 and 60 years of age (164/177), and half declared a previous or current male partner (MSM donors) (77/162, data not available for 15 donors; Figure). Although the number of donors with syphilis infection increased from 20 cases in the first half of 2020 to 57 cases in the first and second half of 2021, no increase in recent syphilis infections was observed (Table 2). Similarly, the number of MSM donors with syphilis increased, but a change in the number of recent infections was not noted. 

**Table 2:** PrEP use among syphilis-positive male donors, England, 2018–2021 (n = 226)

	2018–2019^a^	2020/I	2020/II	2021/I	2021/II
All syphilis-positive males	49	20	43	57	57
Syphilis-positive males with recent infection	13	6	7	10	11
Syphilis-positive males with PrEP use	3	2	1	4	3
All syphilis-positive MSM	13	7	18	20	32
Syphilis-positive MSM with recent infection	4	1	4	4	1
Syphilis-positive MSM with PrEP use	2	2	0	3	1

MSM: men who have sex with men; PrEP: pre-exposure prophylaxis.

^a^ Data for 2018–2019 from Harvala et al. [5].

2020/I = January–June; 2020/II = July–December; 2021/I = January–13 June; 2021/II = 14 June–November. Red box: data collected after introduction of a more individualised donor selection policy in England on 14 June 2021.

Although syphilis is not considered to be a major risk to transfusion recipients in countries with cold storage of blood, it may be transmitted by transfusion of blood collected from asymptomatic donor with acute infection, and it also provides information on donor behaviours to blood services. Donors with a history of syphilis, even if treated, are not eligible to donate in the UK. Furthermore, the level of PrEP/PEP use among syphilis-infected male blood donors remained unchanged following the change in donor selection policy in June 2021. However, a higher proportion of syphilis-infected male donors who declared a previous or current male sexual partner had evidence of PrEP use than those without a history of sex with men (7.6% vs 1.2%). Among donors who did not respond or engage in our post-test discussion, PrEP use was even more likely, although the overall numbers here were small (3/15; Table 3).

**Table 3:** Syphilis-positive male blood donors with evidence of PrEP use and associated sexual behaviours, England, January 2020–November 2021 (n = 177)

	Male donors with syphilis	Evidence of PrEP use	Proportion of donors
Current or previous male sexual partner	79	6	7.6%
No history of sex with men	83	1	1.2%
No or limited response from donor	15	3	20%
Previously treated syphilis infection	50	4	8%

PrEP: pre-exposure prophylaxis.

## Discussion

Our data show that the proportion of PrEP use among syphilis-infected male donors has not changed since the implementation of the individualised risk assessment, but the increase in the number of donors with past syphilis infection might reflect a change in donor selection policy. However, our study provides continuing evidence of blood donor non-compliance seeing as 6% of syphilis-positive male donors in 2020 and 2021 used PrEP (10/177) [4]. This time, PrEP testing was performed anonymously and hence we could not provide these results back to the donors. However, donor notification about detected PrEP could be considered in future studies.

Whereas location in the UK and age do not appear to be associated with PrEP usage, its use was higher in MSM than in heterosexual males. This is consistent with previously published data from surveillance of PrEP users in the UK where 98.5% of PrEP users were male and 93.1% of users identified themselves as gay men [8]. The potential high-risk behaviours associated with PrEP use among blood donors are currently unknown. Similarly, it is unclear if blood donors notice the PrEP-related question in pre-donation questionnaire and answer it fully. 

Although the recent introduction of PrEP has been a very effective way to prevent sexual transmission of HIV among MSM [2], it has alerted the international blood transfusion communities to consider PrEP use as a potential predisposing risk factor for HIV infection and hence a risk for blood safety [9,10]. Non-compliance with the regimen may lead to breakthrough HIV infection with suppressed viral loads and delayed seroconversion, challenging the effectiveness of current blood donation screening using both HIV antibody and RNA testing [11-14]. There have also been documented cases of breakthrough HIV infections despite adherence to prophylaxis [11,12]; use of PrEP/PEP or early treatment modulates the immune response which makes the detection of early HIV infection challenging [13,14]. Behaviours associated with PrEP use might also expose the individuals to other sexually or potentially transfusion-transmitted infections [15]. Our study identified three repeat blood donors with evidence of PrEP/PEP use at time of their latest donation which was discarded due to a confirmed syphilis-positive result. Although we do not know how long they have been taking TFV/FTC, it is possible that they may have had undisclosed risk factors for HIV or other infections also at the time of their previous donation(s).

## Conclusions

Our data highlight undeclared PrEP use among blood donors in England, which is likely to increase when the availability of PrEP continues to improve. Donors taking PrEP/PEP should wait 3 months after the completion of the course before attending to donate blood. It is important to consider clear messaging and pre-donation information of blood donors that ‘undetectable equals untransmissible’ does not apply for transfusion due to large volumes of blood. Detection of PrEP/PEP should be introduced as a marker to monitor non-compliance of blood donors potentially affecting blood safety.

## References

[1] Advisory Committee on the Safety of Blood. Tissues and Organs (SaBTO). Can donor selection policy move from a population-based donor selection policy to one based on a more individualised risk assessment? Conclusions from the For the Assessment of Individualised Risk (FAIR) group. SaBTO; 2020. Available from: https://nhsbtdbe.blob.core.windows.net/umbraco-assets-corp/21001/fair_sabto_20201211.pdf

[2] Sewell J Fakoya I Lampe FC Howarth A Phillips A Burns F Effectiveness of interventions aimed at reducing HIV acquisition and transmission among gay and bisexual men who have sex with men (GBMSM) in high income settings: A systematic review. PLoS One. 2022;17(10):e0276209. 10.1371/journal.pone.0276209 36260550 PMC9581368

[3] Joint United Kingdom Blood Transfusion and Tissue Transplantation Services Professional Advisory Committee (JPAC). Change notification No. 04 – 2019. Pre- and post-exposure prophylaxis for HIV prevention– applies to the whole blood and components donor selection guidelines. Sheffield: JPAC; 2019. Available from https://www.transfusionguidelines.org/document-library/change-notifications/change-notifications-issued-in-2019

[4] Harvala H Reynolds C Ijaz S Maddox V Penchala SD Amara A Evidence of HIV pre-exposure or post-exposure prophylaxis (PrEP/PEP) among blood donors: a pilot study, England June 2018 to July 2019. Sex Transm Infect. 2022;98(2):132-5. 10.1136/sextrans-2021-054981 33782147 PMC8862030

[5] Harvala H Reynolds C Fabiana A Tossell J Bulloch G Brailsford S Lessons learnt from syphilis-infected blood donors: a timely reminder of missed opportunities. Sex Transm Infect. 2022;98(4):293-7. 34380778 10.1136/sextrans-2021-055034

[6] Jackson A Moyle G Watson V Tjia J Ammara A Back D Tenofovir, emtricitabine intracellular and plasma, and efavirenz plasma concentration decay following drug intake cessation: implications for HIV treatment and prevention. J Acquir Immune Defic Syndr. 2013;62(3):275-81. 10.1097/QAI.0b013e3182829bd0 23274933

[7] Nash S Dietrich J Ssemata AS Herrera C O’Hagan K Else L Combined HIV Adolescent Prevention Study (CHAPS): comparison of HIV pre-exposure prophylaxis regimens for adolescents in sub-Saharan Africa-study protocol for a mixed-methods study including a randomised controlled trial. Trials. 2020;21(1):900. 10.1186/s13063-020-04760-x 33121503 PMC7596950

[8] O’Halloran C Owen G Croxford S Sims LB Gill ON Nutland W Current experiences of accessing and using HIV pre-exposure prophylaxis (PrEP) in the United Kingdom: a cross-sectional online survey, May to July 2019. Euro Surveill. 2019;24(48):1900693. 10.2807/1560-7917.ES.2019.24.48.1900693 31796157 PMC6891944

[9] Custer B Quiner C Haaland R Martin A Stone M Reik R HIV antiretroviral therapy and prevention use in US blood donors: a new blood safety concern. Blood. 2020;136(11):1351-8. 10.1182/blood.2020006890 32645148 PMC7483439

[10] Gosbell IB Hoad VC Styles CE Lee J Seed CR . Undetectable does not equal untransmittable for HIV and blood transfusion. Vox Sang. 2019;114(6):628-30. 10.1111/vox.12790 31106848

[11] To KW Lee SS . A review of reported cases of HIV pre-exposure prophylaxis failure with resultant breakthrough HIV infections. HIV Med. 2021;22(2):75-82. 10.1111/hiv.12989 33140556

[12] Markowitz M Grossman H Anderson PL Grant R Gandhi M Horng H Newly acquired infection with multidrug-resistant HIV-1 in a patient adherent to preexposure prophylaxis. J Acquir Immune Defic Syndr. 2017;76(4):e104-6. 10.1097/QAI.0000000000001534 29076941 PMC5792163

[13] de Souza MS Pinyakorn S Akapirat S Pattanachaiwit S Fletcher JLK Chomchey N Initiation of antiretroviral therapy during acute HIV‐1 infection leads to a high rate of nonreactive HIV serology. Clin Infect Dis. 2016;63(4):555-61. 10.1093/cid/ciw365 27317797

[14] Elliott T Sanders EJ Doherty M Ndung’u T Cohen M Patel P Challenges of HIV diagnosis and management in the context of pre-exposure prophylaxis (PrEP), post-exposure prophylaxis (PEP), test and start and acute HIV infection: a scoping review. J Int AIDS Soc. 2019;22(12):e25419. 10.1002/jia2.25419 31850686 PMC6918508

[15] Foster K, Sullivan AK, Hughes G, Simms I, Fifer H, Mohammed H, et al. Addressing the increase in syphilis in England: PHE action plan. London: Public Health England; 2019. Available from: https://www.gov.uk/government/publications/syphilis-public-health-england-action-plan

